# A ratiometric fluorescence probe for the selective detection of H_2_S in serum using a pyrene-DPA–Cd^2+^ complex†

**DOI:** 10.1039/d1ra04277g

**Published:** 2021-07-12

**Authors:** Jihoon Kim, Jinyoung Oh, Min Su Han

**Affiliations:** Department of Chemistry, Gwangju Institute of Science and Technology (GIST) 123 Cheomdangwagi-ro, Buk-gu Gwangju 61005 Republic of Korea happyhan@gist.ac.kr

## Abstract

A ratiometric and selective hydrogen sulfide (H_2_S) detection probe was proposed based on the pyrene-DPA–Cd^2+^ complex through the metal ion displacement approach (MDA) mechanism. While most MDA-based fluorescence probes with paramagnetic Cu^2+^ have focused on the development of a simple turn-on sensor using the broad spectral range of fluorescence enhancement, this ratiometric probe exhibited unchanged monomer emission as a built-in internal reference with an increase in excimer emission with added H_2_S. The demonstrated probe showed a rapid response (within 1 min) and a high sensitivity, with 70 nM as the limit of detection. The selectivity for H_2_S over cysteine, homocysteine and glutathione was confirmed, and reliable fluorescence enhancement, which could be monitored by the naked eye, was observed upon irradiation with handheld UV light. In addition, this detection system was successfully applied to detect H_2_S in human serum without interference from biological molecules.

## Abstract 2

The pyrene-DPA–Cd^2+^ complex is demonstrated as a ratiometric fluorescence probe for selective hydrogen sulfide detection in serum based on a metal displacement approach.

## Graphical abstract

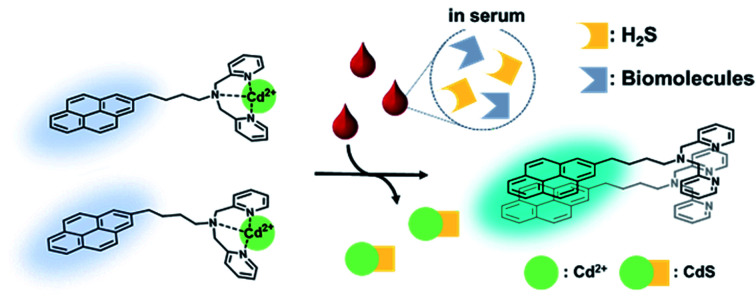

## Introduction

Hydrogen sulfide (H_2_S) is a gaseous signaling molecule that has received attention because of its toxicity for physiological functions in humans.^1,2^ Endogenous H_2_S, which is generated mainly from cysteine (Cys), homocysteine (Hcy), and 3-mercaptopyruvate by enzymatic reactions of cystathionine β-synthase, cystathioine-γ-lyase, and 3-mercaptopyruvate sufurtransferase/cysteine aminotransferase, is involved in various biological processes such as blood pressure regulation, metabolic disorders, neurodegeneration, and inflammation.^3–8^ Therefore, the imbalanced synthesis of endogenous H_2_S is related to some human diseases such as lung infection, Alzheimer's disease, and diabetes.^9–11^ Thus, abnormal levels of endogenous H_2_S in human serum can be used as evidence for such diseases. Considering the importance of H_2_S as a biomarker, various detection methods have been developed for the accurate measurement of changed endogenous H_2_S based on chromatography, electrochemical techniques, colorimetric, and fluorescent chemosensors.^12–15^ Among them, fluorescence detection methods have shown favorable properties such as rapid response, easy sample preparation, and high sensitivity, and various examples have been reported recently.

The currently known methods for fluorescent H_2_S detection were designed by using the chemical properties of H_2_S.^16,17^ These detection methods typically employ four types of probes depending on the recognition mechanism used by them; they may be based on nucleophilicity, reduction, metal coordination, or the metal displacement approach (MDA).^18–21^ MDA is a strategy that exploits fluorescence changes caused by the removal of metal ions, which coordinate to a fluorophore through metal ions and H_2_S complex formation. Owing to the coordination with charged metal ions and the high binding affinity between metal ions and H_2_S, MDA has several advantages over other mechanisms, including good water solubility, rapid response time, and high sensitivity.^22^ Therefore, MDA is a detection mechanism that is appropriate for application in various environments. Although some metal ions, including Cd^2+^, Zn^2+^, Hg^2+^, and Cu^2+^, show very low solubility products (K_sp_) when bound to H_2_S, most proposed examples have focused on paramagnetic Cu^2+^ because fluorescence recovery through CuS complex formation promotes turn-on fluorescence detection after fluorophores are quenched by Cu^2+^ binding; this turn-on detection method displays fewer false positive signals and good distinguishability from the background compared to the turn-off method.^23,24^ Consequently, examples of MDA-based H_2_S detection using other metal ions have been limited to date.

Ratiometric fluorescence probes emit two or more at different wavelengths, and the ratio between the emissions is used to detect the target molecules. Ratiometric probes have unique features that enable self-calibration for the quantification of the target, and they also have built-in correction systems that reduce the environmental interference and enable them to have high signal-to-noise ratios.^25,26^ Therefore, numerous applications of these probes for various analytes have been developed, including H_2_S detection.^27,28^ However, despite the advantages that make MDA among the four typical H_2_S detection mechanisms, this mechanism has not garnered attention as a strategy for the development of ratiometric detection methods except for a little example.^29,30^ This is because quenching the entire spectral range of fluorescence by paramagnetic Cu^2+^ induces only simple fluorescence enhancement instead of ratiometric changes upon addition of H_2_S. In addition, the excessively strong interaction between thiol and Cu^2+^ causes other biothiols such as Cys, Hcy, and glutathione (GSH) to interfere with the reactions and reduce the selectivity of H_2_S detection, as shown in several previous studies.^22^ Thus, it is crucial to develop ratiometric fluorescent probes for the detection of H_2_S using other metal ions that retain the advantages of the MDA-based mechanism and also display great selectivity of H_2_S over other biothiols.

**Scheme 1:**
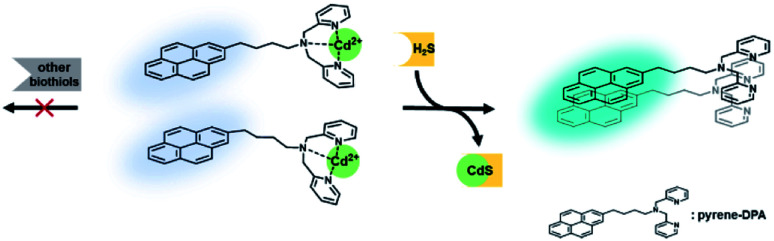
Schematic illustration of ratiometric and selective detection of H_2_S using Cd^2+^ displacement in a pyrene-DPA–Cd^2+^ complex.

In this study, the pyrene-DPA–Cd^2+^ complex was proposed for use in a ratiometric fluorescence probe for H_2_S detection that is selective to other biothiols (Scheme 1). Previous studies performed in our group revealed that coordination of pyrene-DPA with Zn^2+^ decreased excimer emission without changing the monomer emission.^31^ Inspired by this result, it is expected that a complex of pyrene-DPA with a metal ion that has high binding affinity to H_2_S and quenches an excimer emission can be applied to develop ratiometric detection methods based on the MDA using the unchanged monomer as a built-in internal standard. Screening the response of the pyrene-DPA–metal ion complex in the presence of various biothiols, it was confirmed that Cd^2+^, which is in the same group as Zn^2+^, reduced only the excimer emission of pyrene-DPA; in particular, other biothiols apart from H_2_S did not influence any emissions in the pyrene-DPA–Cd^2+^ complex. Thus, the pyrene-DPA–Cd^2+^ complex could be used as a ratiometric probe for selective H_2_S detection over biothiols with high sensitivity and applicability in real serum samples, which were derived from the features of the MDA mechanism.

## Experimental

### Materials and instrumentations

Chemical reagents were purchased from commercial sources (Sigma Aldrich, Alfa Aesar, Duksan, and Tokyo Chemical Industry) and used without further purification. Human serum was purchased from Sigma Aldrich (catalog number: H6914). ^1^H NMR spectra were recorded on a JEOL 400 MHz NMR spectrometer. Fluorescence spectra were measured on Agilent Cary Eclipse fluorescence spectrometer.

### Synthesis of pyrene-DPA and *in situ* generation of pyrene-DPA–metal ion complex

The pyrene-DPA compound was prepared from previous literature of our group.^31^

For *in situ* pyrene-DPA–Cd^2+^ complex, same volume of pyrene-DPA and metal ion solution with same concentration were added to the buffer solution for each measurements and incubated for 5 min. After the incubation, various analytes were introduced and fluorescence or NMR spectra were obtained.

### Screening for selection of an optimal metal ion and pH conditions

To pyrene-DPA–metal ion complex (Cd^2+^, Zn^2+^, Cu^2+^, and Hg^2+^, 20 μM) in buffer solution (20 mM, HEPES, pH 7.4), H_2_S (100 μM) and other biothiols solution (Cys, Hcy, and GSH, 100 μM) was added and emission spectra (*λ*_ex_ = 341 nm) were recorded using fluorescence spectrometer. Pyrene-DPA–Cd^2+^ or Zn^2+^ complex (20 μM) was conducted by pyrophosphate (PPi) solution (50 μM) and fluorescence spectra were measured in the same procedure.

For selection of optimal pH condition, H_2_S and Cys (60 μM) were added to buffer solution (20 mM, pH 5.0 acetate, pH 6.0 MES, pH 7.0, 7.4, and 8.0 HEPES, pH 9.0 Tris) containing pyrene-DPA–Cd^2+^ complex (20 μM) and fluorescence spectra was recorded with excitation at 341 nm.

### Sensitivity for detection of H_2_S

Various concentrations of H_2_S (0, 0.1, 0.2, 0.4, 0.6, 0.8, 1.0, 2.0, 4.0, 6.0, 8.0, 10.0, 12.0, 14.0, 16.0, 18.0, 20.0, 30.0, 40.0, 50.0, 60.0 μM) were added to buffer solution (20 mM, HEPES, pH 7.0) containing pyrene-DPA–Cd^2+^ complex (20 μM) and fluorescence spectra was obtained with excitation at 341 nm. Limit of detection (LOD) was estimated by 3*σ*/*S* using excimer enhancement against monomer emission from 0 to 1.0 μM H_2_S where *σ* is standard deviation of blank and S means slope from titration data for H_2_S detection.

### Selectivity and interference test for detection of H_2_S

Biothiols (60 μM, H_2_S, Cys, Hcy, and GSH) and various anions (60 μM, F^−^, Cl^−^, Br^−^, I^−^, NO_2_^−^, NO_3_^−^, N_3_^−^, CN^−^, HSO_4_, CO_3_^2−^, PO_4_^3−^, and PPi) were added to buffer solution (20 mM, HEPES, pH 7.0) containing pyrene-DPA–Cd^2+^ complex (20 μM) and fluorescence spectra were recorded with excitation at 341 nm. For interference test, H_2_S (20 μM) was introduced to buffer solution (20 mM, HEPES, pH 7.0) containing pyrene-DPA–Cd^2+^ complex (20 μM) and other biothiol or anion (60 μM, Cys, Hcy, GSH, F^−^, Cl^−^, Br^−^, I^−^, NO_2_^−^, NO_3_^−^, N_3_^−^, CN^−^, HSO_4_, CO_3_^2−^, PO_4_^3−^ and PPi), and fluorescence change was measured with excitation at 341 nm.

### Mechanistic study for H_2_S detection based on metal ion displacement

To pyrene-DPA–Cd^2+^ complex (10 mM) in DMSO-*d*_6_, Cd^2+^ (10 mM) in D_2_O was added with or without subsequent addition of H_2_S (10 mM) in D_2_O and ^1^H-NMR was measured in DMSO-*d*_6_ : D_2_O (8 : 2) with 400 MHz NMR spectrometer. The spectra of recorded ^1^H-NMR were compared in particular region.

### Application of H_2_S detection in human serum samples

For preparation of deproteinized human serum sample, ammonium sulfate precipitation and subsequent centrifugal filtration were carried out. And the treated serum solution was spiked with various concentrations of H_2_S (6.0, 10.0, 12.0, 16.0, and 18.0 μM) and incubated for 5 min. The spiked serum sample was added to buffer solution (20 mM, HEPES, pH 7.0) containing pyrene-DPA–Cd^2+^ complex (20 μM) with final 5% serum and obtained fluorescence ratio at 476 and 376 nm was used to estimate found H_2_S concentrations.

## Results and discussion

### Screening for selection of an optimal metal ion and pH conditions

**Fig. 1:**
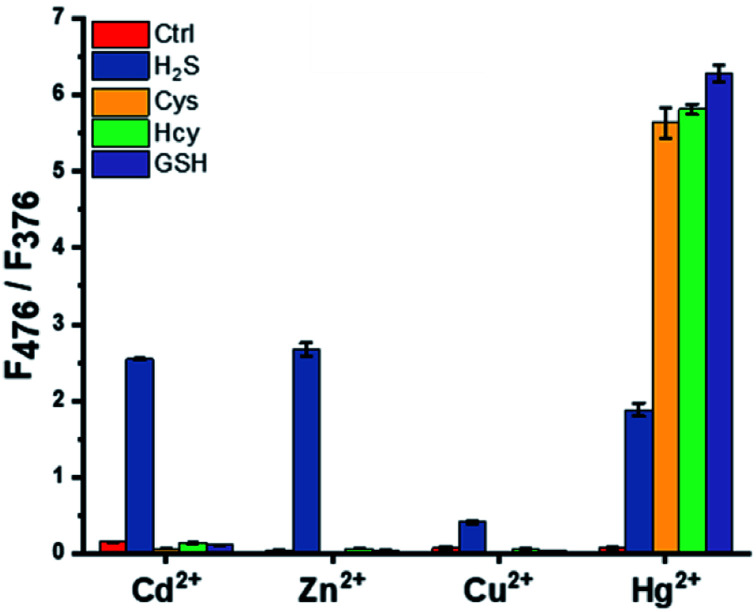
Fluorescence change (*F*_476_/*F*_376_) in the excimer at 476 nm against monomer emission at 376 nm of pyrene-DPA–metal ion complex with excess of biothiols (100 μM) in the buffer solution (HEPES, 20 mM, pH 7.4). [pyrene-DPA–metal ion complex] = 20 μM, *λ*_ex_ = 341 nm.

To develop a ratiometric probe for the selective detection of H_2_S over other biothiols, fluorescence responses through metal ion displacement of pyrene-DPA complexes were compared for various metal ions along with the addition of biothiols. In the case of Cu^2+^ and Hg^2+^, the entire fluorescence-containing monomer and excimer emission were reduced during complex formation between metal ions with pyrene-DPA (Fig. S1†). Therefore, they were not suitable for the development of ratiometric detection probes. Dramatic enhancement of excimer emission at 476 nm against monomer emission at 376 nm (*F*_476_/*F*_376_) was observed only in the pyrene-DPA–Zn^2+^ or Cd^2+^ complex with selectivity for H_2_S over Cys, Hcy, and GSH when excess of biothiols (5 equiv.) was added to the pyrene-DPA–metal ion complex (Fig. 1). Considering that the pyrene-DPA–Zn^2+^ complex was previously used as a probe for the detection of PPi, which is abundant in a biological environment, fluorescence changes under interference from PPi were measured (Fig. S2†).^32^ Unlike the pyrene-DPA–Cd^2+^ complex, the pyrene-DPA–Zn^2+^ complex displayed an increase in excimer emission in the presence of PPi. This property of the pyrene-DPA–Zn^2+^ complex would hinder its application in the detection of H_2_S in real samples. Therefore, the pyrene-DPA–Cd^2+^ complex was chosen as an optimal probe for selective and ratiometric H_2_S detection.

The optimal pH conditions were determined by evaluating the fluorescence responses of the pyrene-DPA–Cd^2+^ complex for H_2_S and Cys because in human serum, the approximate Cys concentration is higher than that of H_2_S, which makes it difficult to distinguish them (Fig. S3†).^33^ At pH values above 7.4, interference of Cys was observed, and this interference increased with the pH. A more drastic change in fluorescence induced by Cys was observed at pH 9.0. At neutral pH, the fluorescence enhancement by H_2_S was still similar to that at basic pH, and interference by Cys was inexistent. As a result, a pH of 7.0 was used in subsequent experiments.

### Sensitivity for detection of H_2_S

**Fig. 2:**
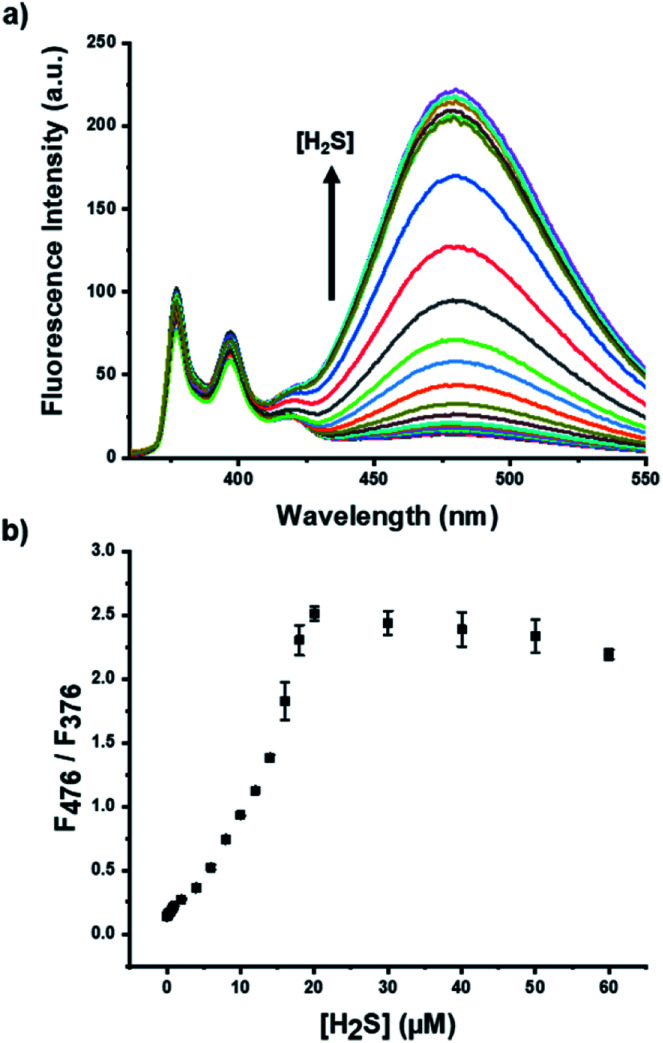
(a) Fluorescence spectra of pyrene-DPA–Cd^2+^ complex obtained 1 min after the addition of various concentrations of H_2_S in the buffer solution (HEPES, 20 mM, pH 7.0); (b) plot of the fluorescence intensity ratio at 476 and 376 nm of pyrene-DPA–Cd^2+^ complex along with the increased H_2_S concentrations. [pyrene-DPA–Cd^2+^ complex] = 20 μM, [H_2_S] = 0, 0.1, 0.2, 0.4, 0.6, 0.8, 1.0, 2.0, 4.0, 6.0, 8.0, 10.0, 12.0, 14.0, 16.0, 18.0, 20.0, 30.0, 40.0, 50.0, and 60 μM, *λ*_ex_ = 341 nm.

The fluorescence change of the pyrene-DPA–Cd^2+^ complex with various concentrations of H_2_S was monitored to verify the sensitivity and the available detection range of the developed probe. The excimer emission at 476 nm upon addition of H_2_S with unchanged monomer emission at 376 nm increased gradually with increasing the H_2_S concentrations (Fig. 2a). The extent of fluorescence change in the excimer against monomer emission as an internal reference exhibited an almost linear relationship with the concentration of H_2_S (Fig. 2b). The largest enhancement was observed with 20 μM of H_2_S because the upper limit of detection was dependent on the concentration (20 μM) of pyrene-DPA–Cd^2+^ complex (Fig. 2b). As expected, the MDA-based mechanism enables rapid detection and high sensitivity; the fluorescence change was observed within 1 min, and LOD was estimated to be 70 nM from titration data (Fig. S4 and S5†).

### Selectivity and interference test for detection of H_2_S

**Fig. 3:**
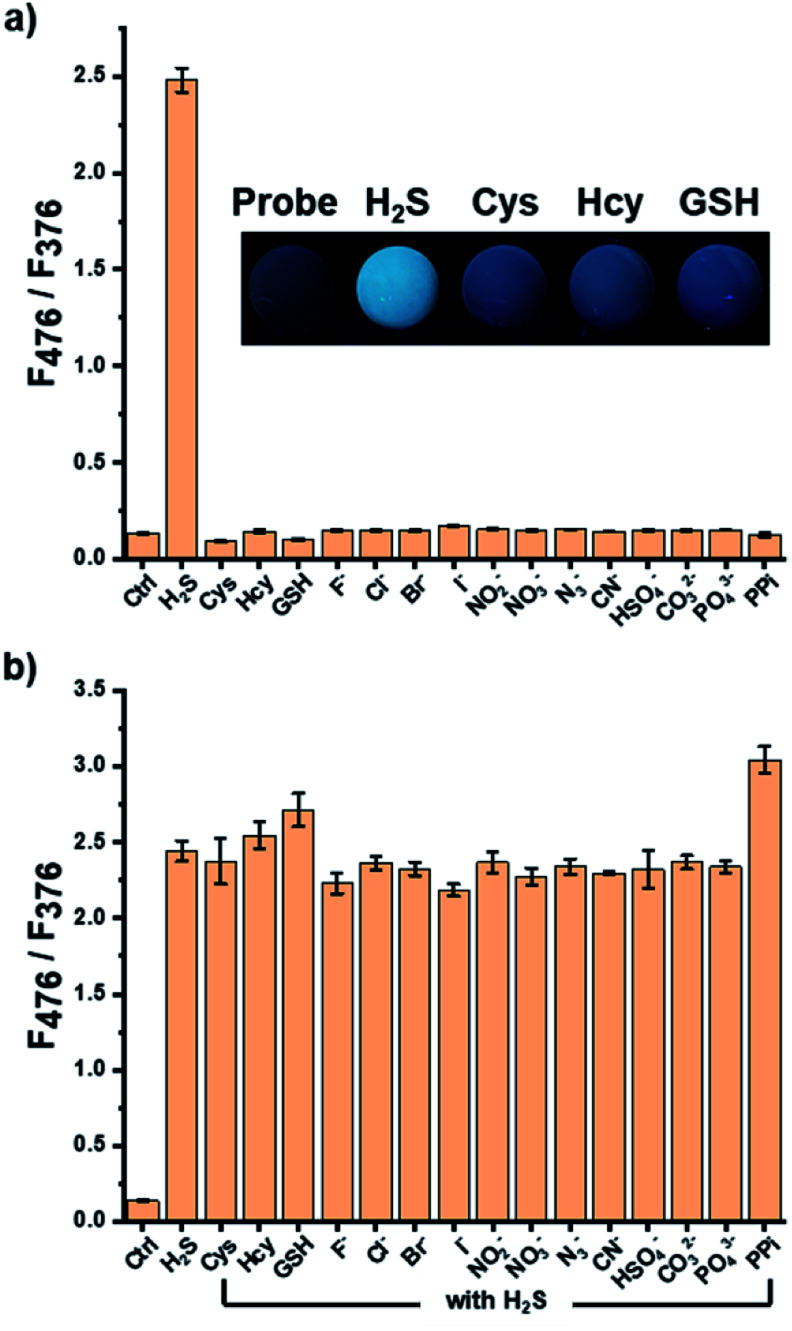
(a) Fluorescence change in the excimer against monomer emission of pyrene-DPA–Cd^2+^ complex in the presence of various biothiols and anions (60 μM), and the photograph of the complex with biothiols under irradiation of handheld UV light (365 nm); (b) fluorescence change of the pyrene-DPA–Cd^2+^ complex by the addition of H_2_S (20 μM) in the presence of biothiol or anion in the buffer solution (HEPES, 20 mM, pH 7.0). [pyrene-DPA–Cd^2+^ complex] = 20 μM, *λ*_ex_ = 341 nm.

In a biological environment, biothiols and various anions can disturb the detection of H_2_S. To investigate the selectivity of the suggested probe, spectral changes of the pyrene-DPA–Cd^2+^ complex in the presence of these compounds were examined. The biothiols, except for H_2_S, did not affect the fluorescence intensity of the pyrene-DPA–Cd^2+^ complex, and the entire fluorescence spectra of the complex did not change upon the addition of various anions (Fig. 3a). In particular, the fluorescence responses with biothiols were photographed under irradiation with handheld UV light, and only H_2_S showed a reliable fluorescence enhancement that can be observed by the naked eye (Fig. 3a). To explore the interference for the probe from biothiols and anions, the detection of H_2_S in the presence of excess biothiols and various anions (3 equiv.) was performed. The coexistence of other biothiols or most anions did not induce a notable interference in the detection of H_2_S, while a slight additional increase in excimer emission with PPi was observed (Fig. 3b). Thus, it is expected that this great selectivity and unimpeded features to detect H_2_S would allow its application in a real serum sample.

### Mechanistic study for H_2_S detection based on metal ion displacement

**Fig. 4:**
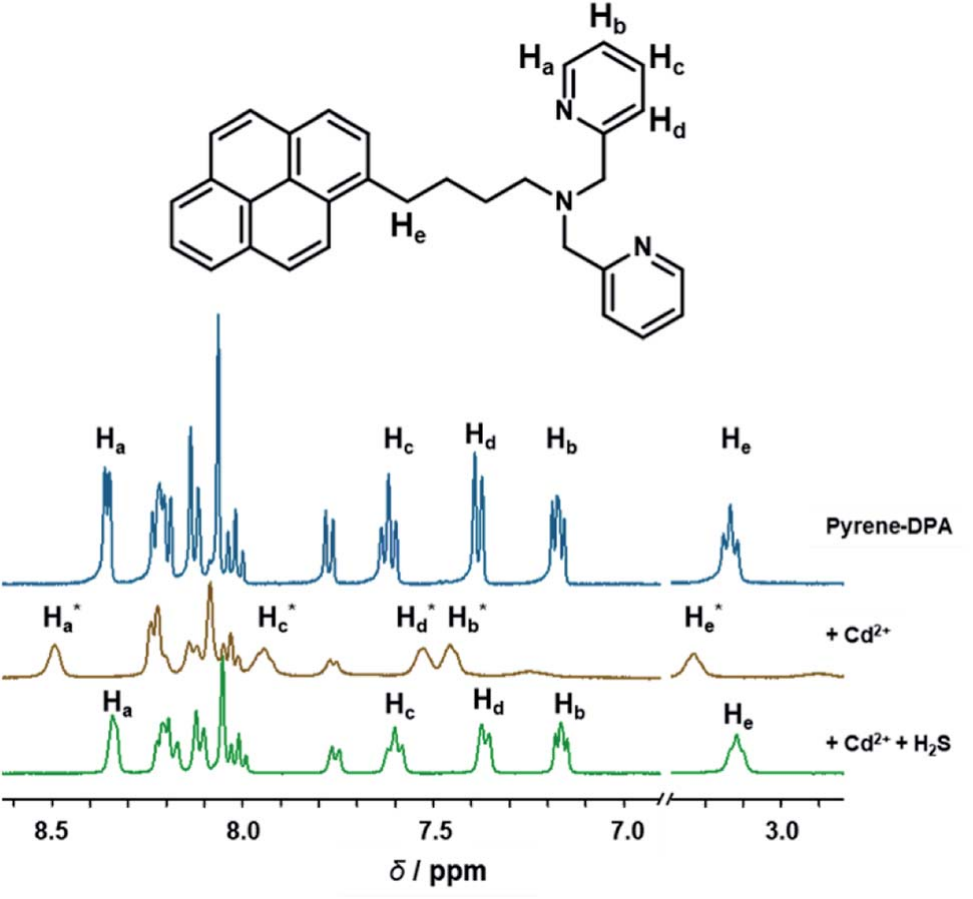
Change of partial ^1^H-NMR spectra (400 MHz) of pyrene-DPA–Cd^2+^ complex (10 mM) in the addition of Cd^2+^ (10 mM) and subsequent H_2_S (10 mM) in DMSO-*d*_6_ : D_2_O (8 : 2).

To evaluate the mechanism for the detection of H_2_S based on the pyrene-DPA–Cd^2+^ complex, ^1^H-NMR spectra along with Cd^2+^ and subsequent H_2_S addition to pyrene-DPA were compared (Fig. 4). The assignment of the proton signal in pyrene-DPA was proposed in a previous report.^34^ The aromatic proton signal (H_a_, H_b_, H_c_, and H_d_) in the DPA group, which is a well-known metal ion binding moiety, was shifted downfield following the addition of Cd^2+^, suggesting that the strong electron-withdrawing Cd^2+^ was coordinated to the DPA group and reduced the electron shielding effect. In succession, the introduction of H_2_S in the pyrene-DPA–Cd^2+^ complex facilitated the recovery of the downfield protons to the initial signal similar to that of pyrene-DPA, regarded as a displacement of Cd^2+^ through the formation of a CdS complex.^35^ In addition, the aliphatic proton (H_e_) near the pyrene unit was moved downfield while coordinating with Cd^2+^ and recovered again with subsequent H_2_S addition, indicating that effective π–π stacking between pyrene was removed by Cd^2+^ addition and recovered by H_2_S addition.^36^ Thus, it could be inferred that the fluorescence excimer emission recovery after quenching on the coordination of Cd^2+^ was caused by the change in distance between pyrene groups due to Cd^2+^ displacement with H_2_S through the MDA mechanism.

### Application of H_2_S detection in human serum samples

The utility of the developed system in a practical sample was confirmed using spiked H_2_S human serum samples. Human serum samples with various concentrations of spiked H_2_S were prepared through a pretreatment process to remove proteins. The fluorescence change caused by the spiked H_2_S was used to estimate the concentrations, and the results were compared with the known values. As summarized in Table 1, the obtained H_2_S concentration from the probe showed recoveries ranging from 94.8% to 110.1%. Moreover, H_2_S detection by the demonstrated detection system was unaffected by most molecules present in human serum, and these results show its potential for accurately and precisely detecting H_2_S in human serum.

**Table 1:** Determination of H_2_S in human serum samples

Samples	Spiked (μM)	Pyrene-DPA–Cd^2+^ complex		
		Found (μM)	Recovery (%)	RSD (%)
Human serum	6.0	5.7	94.8	18.5
	10.0	10.9	109.3	14.4
	12.0	12.6	105.0	16.9
	16.0	17.1	107.1	10.0
	18.0	19.8	110.1	6.4

## Conclusions

We developed a ratiometric fluorescence probe for selective H_2_S detection based on the pyrene-DPA–Cd^2+^ complex, which works based on the MDA mechanism. The increase in excimer emission against a fixed monomer emission, which was used an internal reference, facilitated ratiometric detection of H_2_S, which was previously difficult using the MDA strategy. Using the MDA, rapid response (within 1 min) and high sensitivity (LOD = 70 nM) for the detection of analytes were confirmed. The selectivity for H_2_S over Cys, Hcy, and GSH was exhibited without interference from these molecules, and the selectivity over other biothiols was evaluated through the significant fluorescence enhancement monitored by the naked eye upon irradiation with handheld UV light. Moreover, H_2_S detection in human serum was achieved without interference from other components. Thus, the demonstrated system shows potential for use in practical applications.

## Conflicts of interest

There are no conflicts to declare.

## Supplementary Material

RA-011-D1RA04277G-s001
