# Supplementary material for: A ratiometric fluorescence probe for the selective detection of H2S in serum using a pyrene-DPA–Cd2+ complex
Source: RSC Adv. 2021 Jul 12;11(39):24410–5. doi: 10.1039/d1ra04277g (PMC9036711; doi:10.1039/d1ra04277g)
Supplement: RA-011-D1RA04277G-s001 [file RA-011-D1RA04277G-s001.pdf]

Electronic Supplementary Material (ESI) for RSC Advances.
This journal is © The Royal Society of Chemistry 2021

Electronic Supplementary information

Ratiometric fluorescence probe for the selective detection of H_2S in serum using pyrene-DPA-Cd^{2+} complex

Jihoon Kim,‡ Jinyoung Oh‡ and Min Su Han*

Department of Chemistry, Gwangju Institute of Science and Technology (GIST), Gwangju 61005, Republic of Korea

* Corresponding author. E-mail: happyhan@gist.ac.kr

Fluorescence spectra of pyrene-DPA complex with metal ions

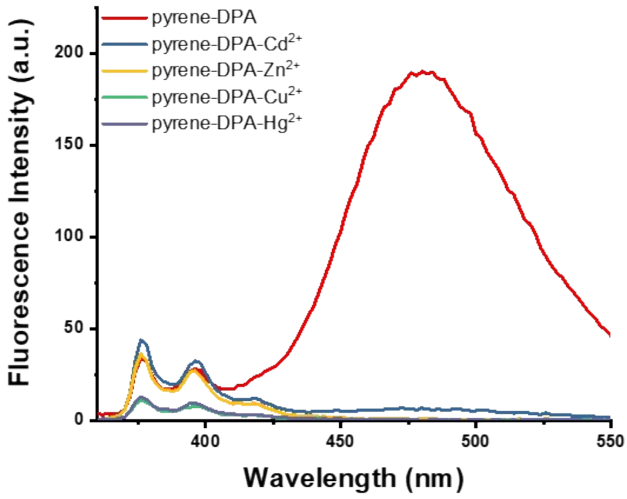

Figure S1 Fluorescence spectra of pyrene-DPA complex with various metal ions (Cd^{2+}, Zn^{2+}, Cu^{2+}, and Hg^{2+}) in buffer solution (HEPES, 20 mM, pH 7.4). [pyrene-DPA] = 20 µM, [metal ion] = 20 µM, λ_{ex} = 341 nm.

Fluorescence change of pyrene-DPA-Cd^{2+} or Zn^{2+} complex with PPi

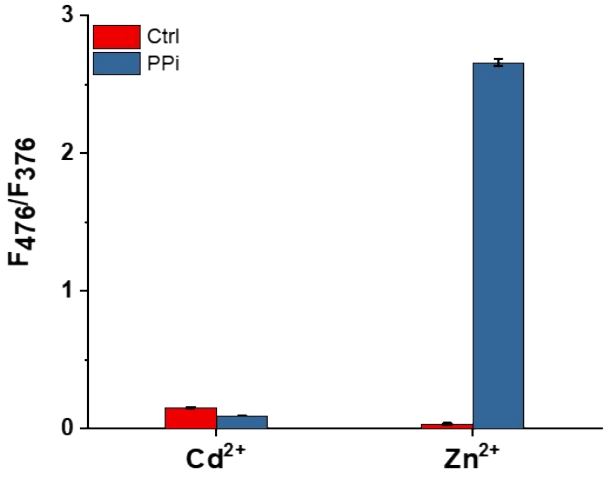

Figure S2 Fluorescence change in excimer against monomer emission of pyrene-DPA-Cd^{2+} or Zn^{2+} complex with PPi (50 μM) in the buffer solution (HEPES, 20 mM, pH 7.4), λ_{ex} = 341 nm.

H_2S and Cys detection in different pH condition

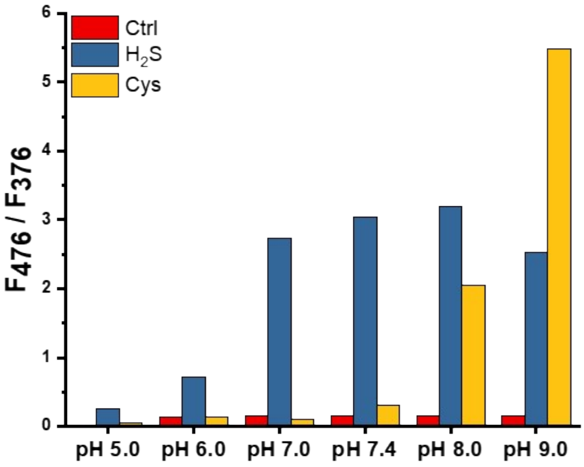

Figure S3 Fluorescence change of pyrene-DPA-Cd^{2+} complex with H_2S and Cys in different buffer solutions (20 mM). [pyrene-DPA-Cd^{2+} complex] = 20 µM, [H_2S] = 60 µM, [Cys] = 60 µM, pH 5.0: Acetate, pH 6.0: MES, pH 7.0, 7.4, and 8.0: HEPES, pH 9.0: Tris, λ_{ex} = 341 nm.

Response time for detection of H_2S by pyrene-DPA-Cd^{2+} complex

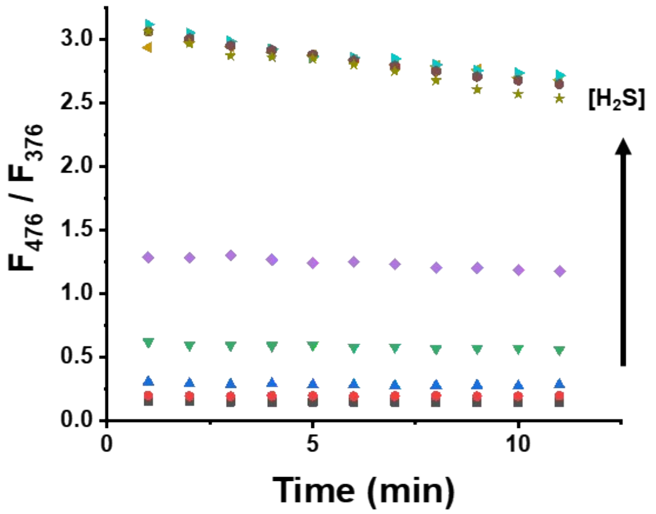

Figure S4 Plot of Fluorescence change of pyrene-DPA-Cd^{2+} complex with various concentrations of H_2S along with time in buffer solution (HEPES, 20 mM, pH 7.0). [pyrene-DPA-Cd^{2+} complex] = 20 µM, [H_2S] = 0.0, 0.5, 1.0, 5.0, 10.0, 20.0, 30.0, 40.0, 50.0 µM, λ_{ex} = 341 nm.

Estimated limit of detection (LOD) for H_2S detection

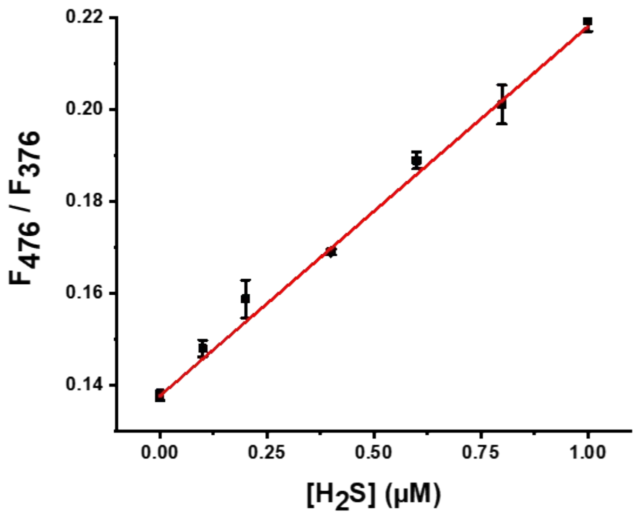

Figure S5 Plot of change of fluorescence ratio at 476 nm and 376 nm against H_2S concentrations (0, 0.1, 0.4, 0.6, 0.8, and 1.0 μM), λ_{ex} = 341 nm.

Stdev. of blank (σ) = 0.00188

Slope (S) = 0.08036

R^2 = 0.9948

Limit of detection (LOD) = 70.18 nM from 3σ/S
